# 135 It’s about time for exercise: the Exercise Participation Explained in Relation to Time (EXPERT) Model

**DOI:** 10.1093/eurpub/ckae114.084

**Published:** 2024-09-26

**Authors:** Sean Healy, Freda Patterson, Stuart Biddle, Dorothea Dumuid, Ignace Glorieux, Tim Olds, Catherine Woods, Adrian Bauman, Aleš Gaba, Matthew Herring, Kaja Kastelic, Ugo Lachapelle, Stella Volpe, Željko Pedisic

**Affiliations:** Department of Physical Education and Sports Science, University of Limerick, Ireland; Department of Physical Education and Sports Science, University of Limerick, Ireland; Department of Physical Education and Sports Science, University of Limerick, Ireland; Department of Physical Education and Sports Science, University of Limerick, Ireland; Department of Physical Education and Sports Science, University of Limerick, Ireland; Department of Physical Education and Sports Science, University of Limerick, Ireland; Department of Physical Education and Sports Science, University of Limerick, Ireland; Department of Physical Education and Sports Science, University of Limerick, Ireland; Department of Physical Education and Sports Science, University of Limerick, Ireland; Department of Physical Education and Sports Science, University of Limerick, Ireland; Department of Physical Education and Sports Science, University of Limerick, Ireland; Department of Physical Education and Sports Science, University of Limerick, Ireland; Department of Physical Education and Sports Science, University of Limerick, Ireland; Department of Physical Education and Sports Science, University of Limerick, Ireland

## Abstract

**Background:**

‘Lack of time’ is consistently the most commonly reported barrier to regular exercise participation. However, this term insufficiently captures the complex and multidimensional nature of time. Recognizing the need for a more comprehensive analysis of ‘lack of time’ as a barrier to exercise, in this presentation the authors introduce the Exercise Participation Explained in Relation to Time (EXPERT) model.

**Purpose:**

To present the temporal (time-related) factors relevant to exercise via the Exercise Participation Explained in Relation to Time (EXPERT) model.

**Methods:**

To develop the EXPERT model, the authors used a rigorous sequential process that comprised the following steps. First, an umbrella literature review of time as barrier, determinant, and correlate of exercise was conducted. Second, a targeted review of existing temporal models was completed. Third, the lead author developed a first draft of the EXPERT model, led a series of focused discussions with members of the authorship team to refine the model, and then conducted a Delphi process to solicit pointed feedback and produce the penultimate model. Finally, this penultimate model was shared with a convenience sample of end-users and experts who informed revisions to make the final EXPERT model.

**Results:**

The final EXPERT model factors includes four categories of factors: (1) Temporal needs and preferences for exercise (an individual’s preferences in relation to when they exercise); (2) Temporal autonomy for exercise (an individual’s autonomy in scheduling their free time); (3) Temporal conditions for exercise (an individual’s actual available time for exercise); and, (4) Temporal dimensions of exercise (an individual’s actual use of time for exercise). These four categories include multiple factors which collectively represent an individual’s preferences for, autonomy over, conditions for, and actual use of time for exercise.

**Conclusions:**

The EXPERT model provides a framework that comprehensively explains the temporal factors that relate to exercise participation. This framework can help guide more nuanced assessments of time as a barrier to exercise and help inform novel approaches to mitigate this pervasive and potent barrier to regular exercise engagement.

